# Management of hypoactive sexual desire disorder in women in the gynecological setting: Number 5 - May 2021

**DOI:** 10.1055/s-0041-1731410

**Published:** 2021-06-28

**Authors:** Lucia Alves da Silva Lara, Sandra Cristina Poerner Scalco, Andréa Cronemberger Rufino, Stany Rodrigues Campos de Paula, Eduardo Siqueira Fernandes, Joice Martins de Lima Pereira, Siglia Sousa de França, Sheila Reis, Suzane Beirão de Almeida, Fabiene Bernardes Castro Vale, Théo Lerner, Yara Maia Villar de Carvalho, Carmita Helena Najjar Abdo, Flávia Fairbanks Lima de Oliveira

**Affiliations:** 1 Faculdade de Medicina de Ribeirão Preto, Universidade de São Paulo, Ribeirão Preto, SP, Brazil.; 2 Universidade do Vale do Taquari, Lajeado, RS, Brazil. Universidade do Vale do Rio dos Sinos, Porto Alegre, RS, Brazil.; 3 Universidade Estadual do Piauí, Floriano, PI, Brazil.; 4 Faculdade de Medicina de Ribeirão Preto, Universidade de São Paulo, Ribeirão Preto, SP, Brazil.; 5 Faculdade de Medicina, Pontifícia Universidade Católica de Minas Gerais, Betim, MG, Brazil.; 6 Sociedade Goiana De Ginecologia E Obstetrícia, Goiânia, GO, Brazil.; 7 Universidade Federal do Acre, Rio Branco, AC, Brazil.; 8 Sociedade Brazileira de Estudos em Sexualidade Humana, Rio de Janeiro, RJ, Brazil.; 9 Santa Casa de Misericórdia de Porto Alegre, Porto Alegre, RS, Brazil.; 10 Faculdade de Medicina, Universidade Federal de Minas Gerais, Belo Horizonte, MG, Brazil.; 11 Faculdade de Medicina, Universidade de São Paulo, São Paulo, SP, Brazil.; 12 Maternidade Cândida Vargas, João Pessoa, PB, Brazil.; 13 Faculdade de Medicina, Universidade de São Paulo, São Paulo, SP, Brazil.; 14 Faculdade de Medicina, Universidade de São Paulo, São Paulo, SP, Brazil.

## Key-points

- Hypoactive Sexual Desire Disorder (HSDD) is the most prevalent sexual dysfunction among women.
- Psychological, biological, behavioral, relational and environmental factors are the main causes of HSDD and should be assessed during HSDD investigation.
- Gynecologists should assess women's sexual problems during routine gynecological consultations.
- Sexual education is the start point of managing HSDD, secondary to psychological, biological, behavioral, relational and environmental factors.
- The multidisciplinary teamwork approach achieves the best results to deal with HSDD.

## Recommendations

- Allow women to talk about sexual concerns in the gynecological setting.
- Offer general sexual education measures (information on physiology of sexual response, genital anatomy, erogenous zones, sexual fantasies, masturbation, bibliotherapy) to improve sexual repertoire. Inform women that sexual desire is an individual dimension that varies from person to person.
- Suggest shared activities and communication techniques for couples in order to bring sex into their everyday routine.
- Suggest a non-hormonal method (copper IUD) for those complaining of HSDD after hormonal contraceptive use. Prescribe topical hormonal therapy, vaginal moisturizers, and lubricants for climacteric women.
- Assess mental health (anxiety, depression) and refer to a competent professional.
- Do not recommend self-masturbation for women victims of sexual abuse as this may arise intrusive thoughts of the sexual abuse.
- For victims of sexual violence, use strategy to redirect their feeling of guilt. Inform that children are victims of the perpetrator as they are unable to give permission or refuse sexual practices.
- Discuss sexual rights and pleasure with women victims of sexual violence. Tell them about their right to have a healthy and pleasurable sexual life just like any other woman, and inform them it was not their fault; it was the perpetrator's crime.
- Counsel for the adoption of a healthy lifestyle, including physical activity, a healthy diet and avoiding smoking, which may contribute to their wellbeing and more receptivity to sexual stimuli.
- Refer to psychotherapy or sex therapy when appropriate.

## Background

Hypoactive Sexual Desire Disorder (HSDD) is defined as the absence or marked reduction of desire or motivation to engage in sexual activity, as manifested by any of the following criteria: *i* ) reduced or absent spontaneous desire (sexual thoughts or fantasies); *ii* ) reduced or absent responsive desire to erotic cues and stimulation; or *iii* ) inability to sustain desire or interest during sexual activity over a period of at least six months that causes distress. 1 A study conducted in 29 countries involving 14,000 women aged 40 to 80 years showed that 26 to 43% of them complained about HSDD. 2 In Brazil, a systematic review showed a prevalence of 11 to 75% of HSDD. 3 4 5 6 7

There are several psychological, biological, behavioral, relational and environmental factors that interfere with sexual desire. 3 The treatment of HSDD requires knowledge about factors that may be associated with the diagnosis of HSDD ( Chart 1 ).

**Chart 1.** Factors associated to sexual complaints in women.

Use of medications and health problems
Current or past psychiatric disorders
History of sexual, physical or emotional abuse
Beliefs and attitudes towards sex
Body image disturbances
Alcohol, drug and substance use disorders
Work-related stress
Relational conflicts

There are personal characteristics, such as motivation for a healthy sex life, level of self-awareness about one's sexual response, knowledge of sexuality, adjustment of interpersonal and dyadic relationships, optimistic personality, among others that define one's ability to modulate the effects of these factors on the sexual function. 4

## What is the scenario regarding the management of HSDD in women in the gynecological setting?

Assessment of the sexual function is often missed in general practitioner settings mostly because physicians have limited knowledge about sexuality and limited academic training in sexual health issues. 5 Women are less likely to report spontaneously their sexual problems to their physicians. However, about 27% of second-year gynecology residents from a tertiary service in human reproduction did not assess the sexual function of their female patients. Moreover, they tend to categorize the sexual response as normal or abnormal according to their own beliefs. 6

## What is the role of the gynecologist in the assessment of HSDD in women?

Gynecologists (Gyn) may provide women with strategies for improvement of the HSDD related to biological conditions, habits, lack of information of sexual health, poor sexual repertoire, and pain penetration disorders. Education measures may help women's understanding of biological and psychological aspects of female sexual response and important aspects of sexual rights and sexual pleasure, as the anatomy of genitalia, erogenous zones, and sexual repertoire. 7 However, when conditions such as sexual violence, dyadic problems, sexual repression and poor mental health are the basis for HSDD, the woman should be referred to psychotherapy and/or a sex therapy (ST).

## What are the most common biologic factors related to HSDD in women in gynecologic routine care?

Several pathological condition may interfere with the female sexual response. We listed some common factors in gynecological routine care that can impair sexual desire in women.

## Arterial hypertension, diabetes, obesity, and metabolic syndrome

Metabolic syndrome and its components like diabetes mellitus, dyslipidemia, obesity, and arterial hypertension may be associated to HSDD and orgasm disturbances, although there is still controversy on this matter. 8 9 The physiopathology of these diseases is related to endothelial dysfunction and frequently associated with anxiety and depression disorders that culminate in high (68.2%) rates of HSDD in women. 10 In postmenopausal women suffering from arterial hypertension, obesity and diabetes some conditions such as the aging process, depression, and the lack of sexual attractiveness are additional risks for sexual dysfunctions. 11

The management of HSDD in women with metabolic syndrome and correlated pathologies of arterial hypertension, diabetes and obesity may comprehend drug prescription 12 and education measures. Personalized sexual pharmacotherapy on-demand such as testosterone, psychoactive agent (bupropion, buspirone), PDE-5 inhibitor (sildenafil) are used to enhance the neuroendocrine balance between sexual excitation and sexual inhibition.

## Psychiatric disorders: anxiety, depression, stress and schizophrenia

Generalized anxiety disorder, panic disorder and stressful experiences offer high risk for HSDD. 13 Chronic stress may increase circulating cortisol levels, which may alter hypothalamus-hypophysis axis (HHA) function, leading to alteration in sexual steroids synthesis. Emotional stress may alter cognition and concentration on sexual stimuli during sexual relations. 13 The odds for women presenting sexual dysfunction increases 4.11 times with depression. 14 Symptomatology associated may be apathy, lack of interest or irritability, restlessness, and sleep disorders, affecting the sex drive. Unsatisfactory sexual activity, in turn, may lead to depression and/or anxiety and relational conflicts. 15

The management of HSDD due to anxiety and depression symptoms involves the use of antidepressants (ADs) that, in turn, may have negative impact on the sexual function, especially serotonin.

Schizophrenia is associated to HSDD in women, and antipsychotic medication and mood stabilizers increase prolactin (PRL) levels, which contribute even more for HSDD in these women. 16 17 The management of HSDD in women with schizophrenia involves referral to a psychiatrist, who may reduce the dosage, change the drug, or add aripiprazole 18 to improve sexual desire.

## Selective Reuptake Inhibitors and Serotonin-Noradrenaline Reuptake Inhibitors

The intensity of the adverse effect in sexual response of women using selective reuptake inhibitors (SSRIs) and serotonin-noradrenaline reuptake Inhibitors (SNRIs) is underestimated and variable. 19 In a population of women with major depressive disorder in remission, HSDD was diagnosed in 64.3% of those using fluoxetine compared to 37.5% of those using escitalopram. 20

Reduction of sex drive induced by antidepressants occurs through a number of mechanisms, including anticholinergic effects, blockade of the 5-HT2C receptor, increase of serotonergic activity, inhibition of nitric oxide production, D2 blockade, testosterone, FSH and LH reduction, sedation, increase in prolactin concentration and through action of neurotransmitters in the parasympathetic system and central nervous system. 15 The use of antidepressants such as bupropion, mirtazapine, trazodone and vilazodone was associated to less negative impact on the sexual function, since they are used as “antidotes” in the treatment of sexual dysfunction induced by SSRIs and SNRIs. 21 Multimodal antidepressants such as vortioxetine at a dosage of 10-20 mg/day have less impact on sexual function, sleep and weight, which favors treatment adherence. 22 Follow-up with a psychiatrist together with a gynecologist is highly recommended. 23 The management of sexual dysfunction induced by serotonin reuptake inhibitors may include discussing the possibility of adding/changing medication by the psychiatrist ( Chart 2 ).

**Chart 2.** Add-back therapy used in sexual dysfunction induced by selective serotonin reuptake inhibitors and selective noradrenergic inhibitor

• Trazodone at a dose of 200-400 mg/day may enhance sexual desire • Bupropion at a dose of 150-30 mg/day may enhance sexual desire, arousal and orgasm • Buspirone at a dose of 30-60 mg/day may enhance sexual desire and orgasm • Mirtazapine at a dose of 30-60 mg/day may enhance orgasm • Refer to psychotherapy and, if necessary, to sex therapy and couples therapy

## Hormonal contraceptives use

Combined oral contraceptives (COC) may reduce sex drive, arousal and sexual pleasure, but do not interfere with sexual satisfaction. 24 Combined oral contraceptives inhibit the HHA interfering with sexual steroids synthesis, which may reduce sexual motivation. 13 In addition, COCs increase the synthesis of the sex hormone binding globulin (SHBG) that binds to testosterone (T), promoting the reduction of circulating testosterone (T), and this may lead to HSDD. Compared to women who use a copper intrauterine device (IUD), those who use medroxyprogesterone acetate quarterly, the vaginal ring or etonogestrel implant are, respectively, at 2.62, 2.53 and 1.60 times more risk of HSDD. 25 Decreased frequency of sexual thoughts and arousability may lead to contraceptive discontinuation. 26 The management of HSDD due to hormonal contraceptives use implies identifying possible confounding factors to assume COC as the real cause of HSDD. Counseling for the adoption of a healthy lifestyle, including physical activity may contribute to the wellbeing and more receptivity to sexual stimuli. 27 A non-hormonal method (copper IUD) may improve HSDD in these cases.

## What are the most common sociocultural and psychological factors related to HSDD in women in the gynecologic routine care?

Several sociocultural and psychological factors such as myths, beliefs, taboos, repression, dyadic problems, sexual violence, and long-term relationship may interfere with the female sexual response. Gyn should assess briefly these conditions in the gynecological routine care.

## Myths, beliefs, taboos, repression

Many women find the educational source of their sexual behavior and practice in religious teachings and the media, although these are not always reliable sources of scientific information. In some cultures, according to religious precepts and morals, sex is a religious duty for women and virginity is a feminine virtue to preserve, women should have sex with their husbands even if they do not wish to do so, they have no right to reach sexual pleasure and should always remain passive when having sex. 28 Thus, some sexual practices that are not aligned with their religious values and beliefs may promote guilt, anxiety, depressive symptoms and relationship difficulties, as well as psychological discomfort, potentially leading to sexual dysfunctions. 29

Unhealthy lifestyle habits and exposure to continuous stressors such as work and caring for small children cause cognitive distraction and negative emotions, which affect sexual desire and arousal. 30 In these cases, managing sexual dysfunctions involves adapting the therapeutic guidance to the religious cultural context and to the patient's belief system. 31 So far, there is no consensus on the management of HSDD resulting from sociocultural factors, mainly secondary to religious beliefs. However, based on expert opinion, it is important to validate the importance of faith and discuss with women about their right to sexual pleasure and the risk for general and sexual health implied in neglecting female sexual pleasure. 7 Techniques such as physical activity 27 and focused attention exercises for stress management decrease cognitive distraction and improve sexual desire/arousal 30 and may contribute to well-being and more receptivity to sexual stimuli. Also, prescribe bibliotherapy and erotic movies to improve sexual repertoire and offer general sexual education measures (erogenous zones, sexual fantasies, clitoral manipulation to achieve orgasm).

## Dyadic factors

Interpersonal aspects such as infidelity, violence (sexual, domestic, psychological and physical), the partner's sexual dysfunction (erectile dysfunction, premature ejaculation), unemployment, poor education, post-traumatic stress, sexually transmitted infections (STIs), low sexual satisfaction, sexual intercourse focused on penetration, and limited sexual repertoire are commonly interrelated with sexual dysfunction. 3 Overall satisfaction with the sex life influences the quality and maintenance of the desire, while rejection produces a negative influence. 32 The complaint of HSDD is common in women whose partners have sexual dysfunctions such as erectile dysfunction (ED) and premature ejaculation. 33 Likewise, when the woman has HSDD, the male partner has more difficulties with his sexual performance, forming a vicious cycle that exacerbates the couple's sexual difficulties. 34 Limited sexual communication between partners leads to both limitation of sexual repertoire and sexual dissatisfaction. 35

## Long-term relationships

Sex drive in long-term relationships may be affected by individual, interpersonal and social factors ( Chart 3 ). 32

**Chart 3.** Factors associated to sexual dysfunctions in long-term relationships

Aspects	Variables that interfere with sexual function
Individual	General and sexual expectations toward the partner, attractiveness, autonomy, affection, commitment, self-esteem, stress, fatigue
Interpersonal	Responsiveness to partner, emotional intimacy with the partner, communication between partners, professional status, sexual compatibility, sexual satisfaction, relationship over time, routine, monotony
Social	Gender expectations, equality, power war, restrictive sexual attitudes, sexual repression

HSDD as a consequence of a long-term relationship is frequently linked to unrealistic expectations, relational routine and sexual discrepancies between couples. 36 Facilitators of sexual function include attractiveness between partners, prioritizing the relationship, maintaining one's individuality, having good communication, emotional intimacy, self-esteem and control over external stressors. 32 Suggest shared activities and communication techniques to the partner aiming to restore the sexual interest and bring sex into everyday routine. 27 Inform women that sexual desire may decline in a long-term relationship associated with the sex routine and aging process. 36

## Marital infidelity

Marital infidelity is defined as a breach of the exclusivity contract established by the couple regarding psycho-emotional and sexual aspects. 37 It refers to the emotional and/or sexual involvement with a person that is not the primary partner in the relationship. Studies on the prevalence of infidelity are long-standing, revealing 20 to 25% of occurrence among married peoples. 38 Infidelity can be caused by several factors especially cultural issues, the couple's sexual dysfunctions, dissatisfaction with the relationship, family patterns, self-esteem issues, sexual abuse in the childhood, and pathological states like hypersexuality. 37 Infidelity may have negative impact on sexual desire in women.

The management of HSDD due to infidelity may include counseling measures such as providing information to women on infidelity rates among couples with the aim to motivate discussion on this issue. In addition, the Gyn may list some factors related to infidelity, like poor quality of the marital relationship, lack of communication and intimacy of couples. Explain that a break in the sexual routine enhances intimacy and that the social interaction of the couple may enhance the dyadic relationship and improve the sexual response. 37 Inform of strategies to improve the sexual repertoire through bibliotherapy and sex aids (toys, literature and erotic movies), stimulate individual or shared sexual fantasies, and innovate the atmosphere for sexual relations. Alternatively refer the woman to a psychotherapist, couple's therapist, and/or sex therapy.

## Sexual violence (sexual abuse and rape)

Sexual assault affects women's general health and may result in HSDD and avoidance of intimate relationships due to lack of confidence, emotional blunting and fear of touching the body related to the traumatic experience. 39 In this case, the woman should be treated by multidisciplinary team, as it is essential to deal with mental health issues. In order to assess sexual problems in these women, the Gyn should ask them to talk about their experience by using open-ended question (would you like to talk about the assault you suffered?).

In addition, the Gyn may validate her sexual complaint related to the assault, and may use strategy to redirect possible feelings of guilt (it is a crime for an adult to sexually molest a child. The child is not aware that the family member or close person is having inappropriate conduct. The child is not capable of consenting or refusing sexual practices. The genital zone has nerve endings that trigger pleasure when touched. The perpetrator of abuse usually attracts the child with gifts, promises and good behavior, but later on, threatens the child, causing fear). Moreover, it should be interesting to inform women on the prevalence of sexual abuse (unfortunately, sexual abuse is very common mainly among girls and is usually perpetrated by a family member or person related to the child). This approach may help women to feel more confident to share their experience related to the trauma. Finally, the Gyn should refer the woman to a psychiatrist or psychotherapy, depending on the level of distress, and later, to a sex therapist.

## How physiological hormones affect sexual desire in women?

### Pre- and post-menopause period

During menopausal transition, endocrine alterations and the genitourinary syndrome of menopause (GSM) may affect the quality of life and cause HSDD. 40 Social conditions or stressors are also important in the decline of sexual desire and should be primarily observed when dealing with menopausal women. 41 Dyspareunia due to hypoestrogenism may cause sexual activity avoidance. 42 In these cases, local therapy may be suggested. 43

In postmenopausal women, the use of transdermal testosterone may improve HSDD. 44 Oral or injected testosterone is not recommended and the use of dehydroepiandrosterone (DHEA) to treat HSDD does not seem effective. In women with genitourinary syndrome, topical estrogen is recommended according to eligibility criteria. 41 Alternatively, the use of vaginal moisturizers and lubricants is safe to improve sexual pain in these women.

### Pregnancy-puerperal period

In this period, the changes in the female sexual response affect 40 to 70% of pregnant women. 45 A reduction in the sexual frequency and satisfaction has been shown as a consequence of fear of hurting the fetus, impaired body image, dissatisfaction with the partner and mood problems in this phase, 46 as well as because of changes in the focus of attention and also due to the woman's tiredness. 47 The second quarter of pregnancy is the most favorable period for the female sexual function. 48 In the puerperal period, 58.3% of Brazilian nursing mothers complained of a reduction in sexual frequency and poor communication with the partner. 49 Dyspareunia may affect 41% of women after vaginal delivery and 2% after cesarean delivery 50 , which may cause discomfort and HSDD. The management of HSDD in the pregnancy-puerperal period is based mainly in counseling measures 7 51 , the prescription of sexual educational strategies for women's management of HSDD (erogenous zones, sexual fantasies, clitoral manipulation to achieve orgasm), and the use of lubricants and moisturizers to improve pain during penis-vagina penetration.

## Final considerations

HSDD is prevalent in women and there are several psychological, biological and sociocultural factors related to this condition. Gynecologists should receive training to manage this problem in the gynecologic setting. Several factors related to HSDD cannot be addressed by the Gyn and women should be referred to a specialist for an adequate management of HSDD. However, the Gyn may promote sexual health through counseling, education strategy and specific measures to treat endocrine problems related to HSDD in women.

National Specialty Commission for Sexology of the Brazilian Federation of Gynecology and Obstetrics Associations (FEBRASGO)

President:

Lúcia Alves da Silva Lara

Vice-President: Sandra Cristina Poerner Scalco

Secretary:

Flavia Fairbanks Lima de Oliveira

Members:

Andrea Cronemberger Rufino

André Marquez Cunha

Carmita Helena Abdo

Eduardo Siqueira Fernandes

Fabiene Bernardes Castro Vale

Gerson Pereira Lopes

Joice Martins de Lima Pereira

Siglia Sousa de França

Stany Rodrigues Campos de Paula

Suzane Beirao de Almeida

Théo Lerner

Yara Maia Villar de Carvalho
